# Red-phosphorus-impregnated carbon nanofibers for sodium-ion batteries and liquefaction of red phosphorus

**DOI:** 10.1038/s41467-020-16077-z

**Published:** 2020-05-20

**Authors:** Yihang Liu, Qingzhou Liu, Cheng Jian, Dingzhou Cui, Mingrui Chen, Zhen Li, Teng Li, Tom Nilges, Kai He, Zheng Jia, Chongwu Zhou

**Affiliations:** 1 Department of Electrical Engineering, University of Southern California, Los Angeles, California 90089 USA; 2 Department of Materials Science and Engineering, University of Southern California, Los Angeles, California 90089 USA; 3 Department of Mechanical Engineering, University of Maryland, College Park, Maryland 20742 USA; 4 Department of Chemistry, Technical University of Munich, 85748 Garching b. München, Germany; 5 Department of Materials Science and Engineering, Clemson University, Clemson, South Carolina 29634 USA; 6 Department of Engineering Mechanics, Zhejiang University, Hang Zhou, Zhejiang 310058 China

**Keywords:** Energy science and technology, Materials science, Nanoscience and technology

## Abstract

Red phosphorus offers a high theoretical sodium capacity and has been considered as a candidate anode for sodium-ion batteries. Similar to silicon anodes for lithium-ion batteries, the electrochemical performance of red phosphorus is plagued by the large volume variation upon sodiation. Here we perform in situ transmission electron microscopy analysis of the synthesized red-phosphorus-impregnated carbon nanofibers with the corresponding chemo-mechanical simulation, revealing that, the sodiated red phosphorus becomes softened with a “liquid-like” mechanical behaviour and gains superior malleability and deformability against pulverization. The encapsulation strategy of the synthesized red-phosphorus-impregnated carbon nanofibers has been proven to be an effective method to minimize the side reactions of red phosphorus in sodium-ion batteries, demonstrating stable electrochemical cycling. Our study provides a valid guide towards high-performance red-phosphorus-based anodes for sodium-ion batteries.

## Abstract (web summary)

Red phosphorus is a promising anode for Na-ion batteries but suffers from large volume change upon cycling. Here the authors show a red-phosphorus-impregnated carbon nanofiber design in which the sodiated red phosphorus is featured by a “liquid-like” behavior and ultra-stable electrochemical performance is realized.

## Introduction

Lithium-ion chemistry has been extensively implemented to store energy and deliver power for cellular and automotive applications, owing to its remarkably high energy density and long cycle life^1–3^. However, the potential environmental impacts and the unevenly distributed lithium deposits have sparked an exploration of alternative energy storage strategies, such as sodium-ion chemistry^4–8^. The anode part has been considered as the major stumbling block of sodium-ion chemistry because the intercalation of Na ions into graphite is thermodynamically unfavourable; this problem is intuitively associated with the larger ionic radius of the sodium ions, while the reaction energetics may also play a more deterministic role since larger alkali metal ions can exhibit reversible intercalation^6^. Nevertheless, it implies that we should not simply adopt the recent strategies for lithium-ion battery electrode materials directly to sodium-ion chemistry. The commercialization of sodium-ion batteries demands appropriate options of anode materials. Phosphorus (P) is one of the most abundant elements in the continental crust and oceans, and its specific capacity of 2596 mAh g^−1^ offers much promise to sodium-ion batteries. The white phosphorus (P_white_) is extremely toxic and reactive, and the production method of black phosphorus (P_black_) is costly and poorly scalable, promoting red phosphorus (P_red_) into a better position for sodium-ion storage^9,10^.

Just like silicon anodes for lithium-ion batteries, P_red_ anode was believed to be suffering from its insulating nature and huge volumetric expansion upon ion intercalation. Borrowing the ideas from the significantly improved performance of silicon anodes during the past two decades, P_red_ was usually bundled with conductive scaffold, such as carbon nanotubes^11–13^, graphene^14–16^, and other carbonaceous materials^17–21^ to solve the above issues; and thus many researchers, including us, assumed that P_red_ would have the same major problems as silicon: >300% volume expansion from P_red_ to Na_3_P would cause severe pulverization of the particles and lead to the loss of active material. Although the electrochemical performance of P_red_ has been greatly improved, evidence revealing the fundamental mechanism of its capacity decay is still unclear and the long-term cycling performance remains unsatisfied.

To investigate the sodiation process and the capacity decay mechanism of P_red_, we synthesize P_red_-impregnated carbon nanofiber composite (P_red_@CNF), whose one-dimensional structure makes it ideal to observe the sodiation behaviour of P_red_, and the highly conductive CNF shell can serve as electron pathway to improve the charge transfer kinetics of P_red_. Our in situ transmission electron microscopy (TEM) analysis on the sodiation process of P_red_@CNF reveals that P_red_ particles become softened during the sodiation process with a “liquid-like” mechanical property, and the corresponding chemo-mechanical simulation indicates that P_red_ should suffer much less from the volume variation and fracture than the silicon anodes in lithium-ion chemistry. Based on the above observation and simulation, we conclude that the main cause of the P_red_ capacity degradation should be the side reactions that occur during the formation of highly reactive sodiated phosphorus compounds. Furthermore, the designed encapsulation strategy of the synthesized P_red_@CNF composite has been proven to be an effective route to avoid the unfavourable side reactions of P_red_ anode during long-term cycling: the P_red_@CNF anode can deliver ~1850 mAh g^−1^ specific capacities over 500 cycles at 0.1 A g^−1^ current density, and >1000 mAh g^−1^ capacity over 5000 cycles at 1 A g^−1^ rate.

## Results

### As-synthesized material structures

**Fig. 1:**
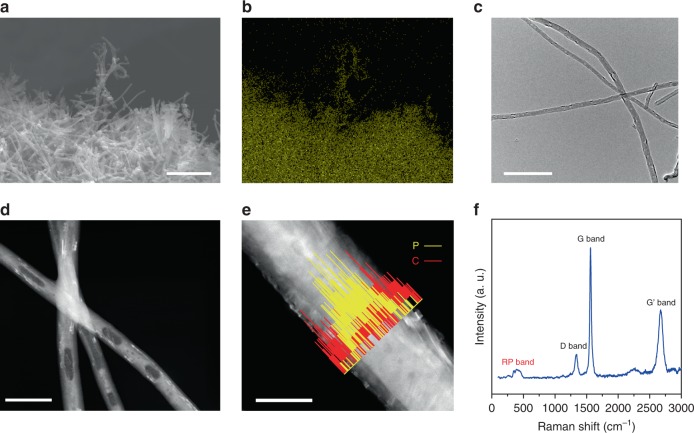
Characterization of as-synthesized P_red_@CNF composite. **a** SEM image of the P_red_@CNF composite. Scale bar is 1 μm. **b** EDS elemental map of the phosphorus concentration in **a**. **c**, **d** TEM and STEM images of the P_red_@CNF composite. Scale bars are 500 nm and 200 nm for **c** and **d**, respectively. **e** EDS line-scan profiles of phosphorus and carbon. Scale bar is 100 nm. **f** Raman spectra of the as-synthesized P_red_@CNF composite.

P_red_@CNF was synthesized through the vaporization–condensation method. First, P_red_ was deposited onto and into the CNFs in a sealed quartz ampoule under vacuum, and then the exceeding P_red_ coated outside of the CNFs was removed with a flash-heat process in an argon-flowed tube furnace, as described in the Methods section and the process flow in Supplementary Fig. 1a. The scanning electron microscopy (SEM) image of the P_red_@CNF composite is exhibited alongside the energy-dispersive analytical X-ray spectroscopy (EDS) profile of phosphorus elemental distribution in Fig. 1a and 1b, where CNFs can be visualized under SEM, and the elemental mapping profile of phosphorus is in a good agreement with the CNF morphology, indicating that most of the phosphorus has been confined inside CNFs. The TEM (Fig. 1c), corresponding high-angle annular dark-field scanning transmission electron microscopy (HAADF-STEM; Fig. 1d), and the EDS mapping images (Fig. 1e) show that the diameter of CNFs is ~100 nm, and the P_red_ segments were encapsulated in CNFs, forming the core-shell nanostructure. The highly graphitized CNF shell provides excellent electronic conductivities and thus improves the charge transfer of P_red_ segments, and the free space between P_red_ segments can further accommodate the substantial volume expansion. Additional SEM and TEM characterization at different synthesis stages can be found in Supplementary Fig. 1b–j. In Supplementary Fig. 1h and 1i, the P_red_ segments and the core-shell nanostructure can be visualized in most of the CNFs under SEM at an acceleration voltage of 15 kV, indicative of the good uniformity of synthesized P_red_@CNF sample. The Raman spectra of the P_red_@CNF composite is exhibited in Fig. 1f, where the P_red_ band ranging from 300 to 500 cm^−1^ is observed, and the characteristic peaks of CNF such as D, G, and Gʹ bands can be identified^22,23^. In both Raman (Fig. 1f) and X-ray photoelectron spectroscopy (XPS) results (Supplementary Fig. 2a), no obvious P–C bond signal can be detected^24^, and the carbon 1 s XPS profiles of pristine CNF and synthesized P_red_@CNF are almost identical, indicating that the bond structure is not significantly affected by the heat treatment process. In the XPS profile of phosphorus 2p edge (Supplementary Fig. 2b), a small P–O bond peak can be attributed to air exposure during the sample transfer process. The Fourier-transform infrared spectroscopy (FTIR) profiles of CNF, P_red_, and the as-prepared P_red_@CNF are exhibited in Supplementary Fig. 3, where the P=O bond signal can be detected from both P_red_ and P_red_@CNF samples; however, no obvious P–O–C or P–C bond signal can be observed from the P_red_@CNF sample. The thermogravimetric analysis (TGA) results of each three bunches of samples (before and after the flash-heat treatment) were exhibited in Supplementary Fig. 4, showing good repeatability of the designed synthesis. The weight percentage of the P_red_ in the P_red_@CNF composite is ~35%.

### In situ TEM experiments and chemo-mechanical simulation

**Fig. 2:**
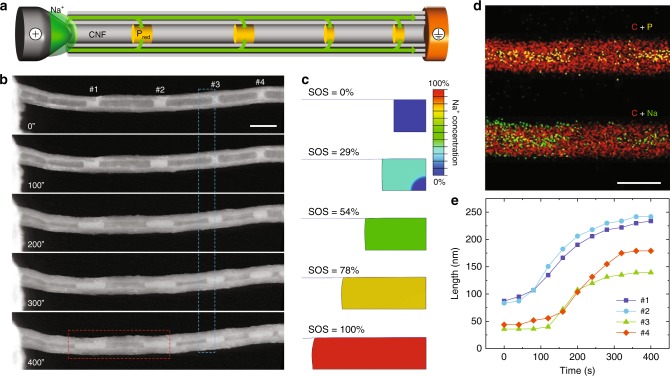
In situ TEM experiment with the relevant analysis. **a** Schematic diagram showing the in situ TEM experimental setup of a dry electrochemical cell. **b** STEM image series captured in real-time showing P_red_ volume expansion during sodiation. Scale bar is 200 nm. **c** Simulated SOS according to the P_red_ morphological evolution of the region labelled with the blue rectangle in **b**. **d** EDS maps of phosphorus (green), sodium (yellow), and carbon (red) elements of the region labelled with the red rectangle in **b**. Scale bar is 200 nm. **e** The length change of four P_red_ segments marked in **b** as the function of time during the sodiation process.

Real-time observation of the sodiation process is critical to investigate the electrochemical reaction mechanisms. The architectural core-shell nanostructure ensures the electronic conductivity of the composite in the TEM electrochemical cell, and the one-dimensional space confined by the CNF shell eases the observation and measurement, at the same time. A dry-format electrochemical cell setup was employed for the in situ study of the sodiation^25–27^, as illustrated in Fig. 2a, where the P_red_@CNF composite and Na metal works as the working and counter electrodes, and the native Na_2_O coating formed on Na metal surface serves as the solid-state electrolyte. The cropped time-lapse STEM image series are exhibited in Fig. 2b and Supplementary Movie 1, where the sodiation of four P_red_ segments inside a single CNF started almost spontaneously, indicative of the fast sodium-ion conduction through CNF shells. The sodiation process was accomplished within 400 s, demonstrating the good sodiation rate boosted by the P_red_@CNF composite architecture. The P_red_ segments accommodate the large volume expansion by longitudinal expansion along the inner space of CNF, without causing apparent deformation or cracking of the stiff CNF shell. The drastic extension of P_red_ segments shown in Fig. 2b reveals a “liquid-like” material behaviour of P_red_ under sodiation: as sodiation advances, P_red_ may yield at low yielding stress and exhibit decreasing stiffness (i.e., softening), enabling the P_red_ to flow plastically within the CNF as the fluid does. It is worth noting that the observed “liquefaction” phenomena are from the intrinsic nature of P_red_, but not due to the electron beam-induced heating effect^28,29^, which was not observed in other allotropes, such as P_black_ (ref. ^30^). No obvious sodiation reaction front or phase boundary was observed in the experiment. The morphology evolution of P_red_ segments with mechanical softening behaviour was modelled as a two-step process via finite element package ABAQUS^31–35^. We defined the state of sodiation (SOS) with SOS = 0% being the pristine unsodiated state and SOS = 100% the fully sodiated state of the P_red_. Simulated P_red_ morphologies corresponding to SOS of 0%, 29%, 54%, 78%, and 100% are presented in Fig. 2c, which is in a good agreement with the longitudinal flow of P_red_ segments observed in the in situ TEM experiments (for example, see segment #3 in Fig. 2b for comparison, as marked with the blue rectangle). Moreover, the formation of rounded convex edges of P_red_ during sodiation is captured by in situ STEM imaging and reproduced by our modelling, indicative of the mechanical interaction between the P_red_ segment and CNF. In the simulation results of Supplementary Fig. 5, it is also predicted that a hard P_red_ segment without sodiation-induced mechanical softening can deform the CNF in the radial direction significantly, and does not flow as readily as observed in the experiments, indicative of the softening effect on P_red_ from another perspective. The EDS elemental mapping profiles of carbon, sodium, and phosphorus elements are displayed in Fig. 2d, in which the distribution of sodium and phosphorus inside the CNF hollows reached a good agreement, indicative of a complete sodiation process forming sodium phosphides. The substantial volume expansion analysis of the P_red_ segments is exhibited in Fig. 2e, where an average longitudinal expansion along CNF of ~330% was confirmed.

**Fig. 3:**
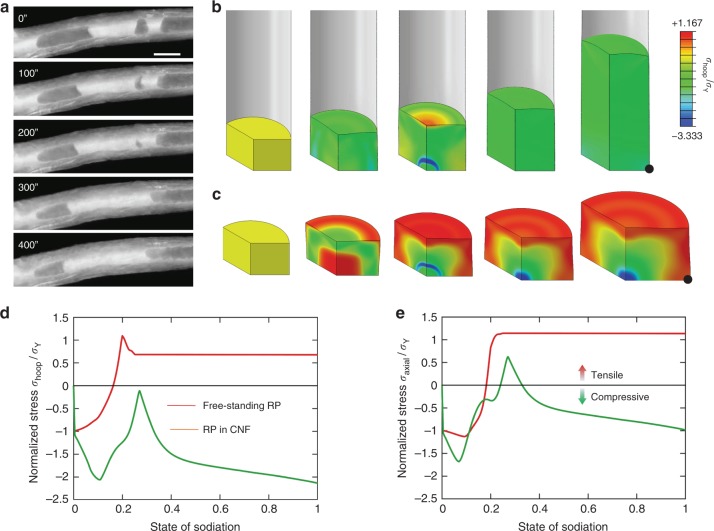
“Liquefication” phenomena with the relevant simulation analysis. **a** STEM image series captured in real-time showing two P_red_ segments merging together during sodiation. Scale bar is 100 nm. Simulated hoop stress distribution of **b** P_red_@CNF and **c** a freestanding P_red_ particle. **d** Hoop stress evolution and **e** axial stress evolution at the hot spot (labelled by black) of P_red_@CNF and a freestanding P_red_ particle during sodiation.

Intriguingly, in another set of the cropped time-lapse STEM images depicted in Fig. 3a and Supplementary Movie 2, just like two liquid droplets, two P_red_ segments deformed and merged during the sodiation expansion, which confirms the sodiation-induced softening effect of P_red_. Usually, P_red_ with polymeric structure can be considered as an intermediate phase between P_white_ (soft and waxy due to the molecular structure, and reactive because of the high angular strain of the molecule) and violet phosphorus (P_violet_, stable due to the monoclinic structure). In this approach, considering that the vaporization–condensation synthesized P_red_ has relatively weak molecular interactions and loose crystal structure, the sodiation-induced bond breaking may partially convert polymeric P_red_ into intermediate phases with similar properties to the soft and waxy P_white_, and introduce the lubricating and softening effects with the “liquid-like” mechanical property.

The modelling results further reveal the mechanical constraint effect provided by the CNF on the stress mitigation in the P_red_ during sodiation. Tensile stress on the material surface is the major driving force for electrode fracture and capacity fading during electrochemical cycling. For example, it is the tensile hoop stress (the normal stress in the circumferential direction, Supplementary Fig. 6a) that causes the fracture of nanoparticle and silicon nanowire anodes in lithium-ion batteries. Fig. 3b shows the evolution of hoop stress as well as the morphology of a P_red_ segment encapsulated by a CNF shell, with the stress level denoted by the colour contour. The lateral expansion of the P_red_ segment is strongly confined by the stiff CNF (Supplementary Fig. 7a–d), driving the P_red_ to plastically flow along the longitudinal direction of the CNF. Consequently, the resulting stresses in the CNF-enclosed P_red_ segment are largely compressive, which prevents crack initiation in the P_red_ segments. As illustrated in Fig. 3c, in contrast to the CNF-encapsulated P_red_ segment, higher tensile stresses are apparent near the segment surface of a freestanding P_red_ segment, largely due to the pushing-out effect during sodiation and, most importantly, the absence of the mechanical constraint provided by the CNF shell. As evident in Fig. 3d, hoop stress at a hot spot (labelled by the black dot in Fig. 3b, c) on the P_red_ segment surface remains compressive during the sodiation process, which is in stark contrast to the high tensile stress developed at the hot spot in the segment without any confinement. The CNF shell not only reduces the hoop stresses in the segment, but also plays an important role in mitigating the axial stresses (the stress in the longitudinal direction, as illustrated in Supplementary Fig. 6b). As revealed in Fig. 3e and Supplementary Fig. 6c, the axial stress on the same hot spot is largely compressive in the CNF-encapsulated P_red_ segment during sodiation, while the freestanding segment constantly undergoes tensile axial stress that may cause fracture and capacity fading (Supplementary Fig. 6d). Based on the analysis above, the structural integrity of the P_red_ segment can be preserved by the designed strategy, which is beneficial for the cycling stability.

### Electrochemical tests

The synthesized P_red_@CNF composite was fabricated into freestanding films through vacuum filtration, without any polymer binder or conductive additives, as shown in the optical image of a pouched film electrode presented in Supplementary Fig. 8. The electrochemical performance of the P_red_@CNF anodes was examined in half-type coin cells with sodium metal as counter electrodes. NaPF_6_ (1.0 mol L^−1^) in an ethylene carbonate (EC)–diethyl carbonate (DEC) solution with fluoroethylene carbonate (FEC) additive (10% by volume) was employed as the electrolyte. Because no obvious P–C bond signals were observed in Raman and XPS results, the specific capacity and current density values are calculated from the mass of phosphorus only.

**Fig. 4:**
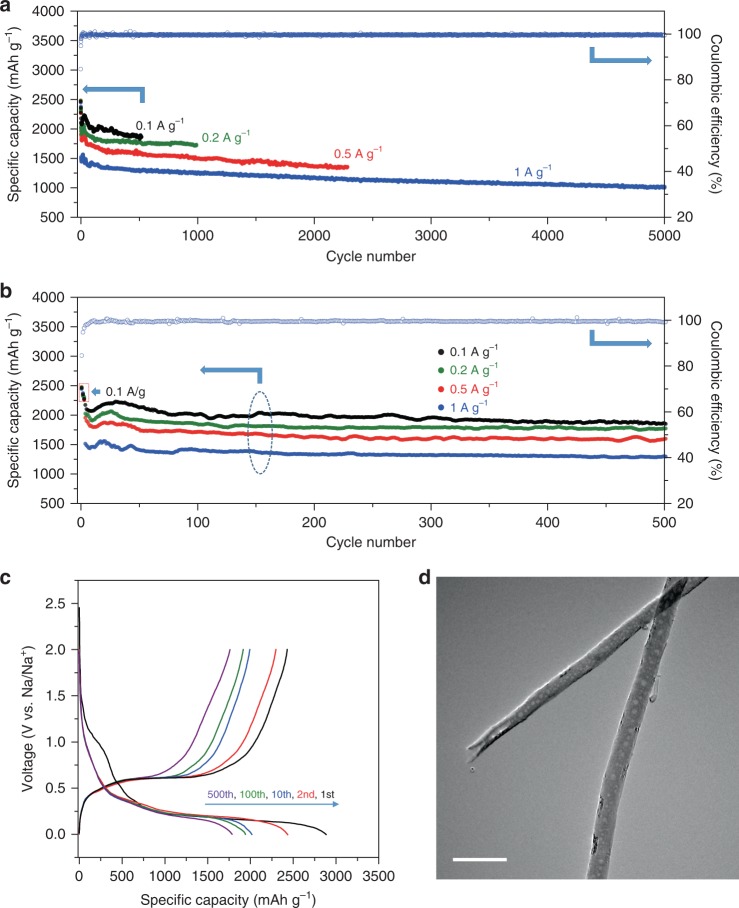
Electrochemical cycling performance and post-cycling TEM image. **a**, **b** Reversible de-sodiation specific capacity of the P_red_@CNF anodes at different current densities with cycle numbers up to 5000 and 500. The charge and discharge current densities of four anodes were 0.1 A g^−1^ for the first three cycles, and 0.1, 0.2, 0.5, and 1 A g^−1^ for later cycles marked in black, green, red, and blue, respectively. Each specific de-sodiation capacity data point is presented as a solid dot against the left axis, and Coulombic efficiency at 1 A g^−1^ current density is presented as hollow circles against the right axis. **c** Sodiation and de-sodiation potential profiles at 0.1 A g^−1^ current density (black curve in **a** and **b**), marked with representative cycle numbers. **d** Post-cycling TEM image of P_red_@CNF after 5000 cycles at 1 A g^−1^ current density. Scale bar is 200 nm.

The cycling stability tests of our P_red_@CNF anodes were performed using galvanostatic charge and discharge with a voltage window of 0.01–2.0 V at 0.1, 0.2, 0.5 and 1 A g^−1^ current densities, with cycle numbers up to 522, 1000, 2284, and 5000, as exhibited in Fig. 4a (1–5000 cycles) and 4b (1–500 cycles) marked with different colours, respectively. The Coulombic efficiency at 1 A g^−1^ current density is presented as hollow circles against the right axis, and efficiencies at other current densities are plotted in Supplementary Fig. 9. In order to form stable solid electrolyte interface (SEI) films with FEC additive, all four anodes were cycled at 0.1 A g^−1^ current density for the first three cycles, as marked with the blue rectangle in Fig. 4b. At 0.1 A g^−1^ current rate, the P_red_@CNF anode presents initial specific charge capacities of 2464.5, 2356.8, and 2275.4 mAh g^−1^ for the first three cycles, and then the capacity decreased from 2177.3 to 1862.5 mAh g^−1^ from the 4th to 522nd cycle with a decay rate of ~0.03% per cycle. The other three anodes also present excellent capacity retention during long cycling: 2032.3 to 1721.1 mAh g^−1^ from the 4th to 991st cycle at 0.2 A g^−1^ current density, 1961.6 to 1361.2 mAh g^−1^ from the 4th to 2284th cycle at 0.5 A g^−1^, and 1522.4 to 1019.5 mAh g^−1^ from the 4th to 5000th cycle at 1 A g^−1^. The superior long cycling performance of P_red_@CNF anode not only confirms that the capacity degradation of P_red_ anode mainly comes from the side reactions occuring at the electrode/electrolyte interfaces, but also suggests that our encapsulation strategy is crucial to optimize the electrochemical stability of P_red_ anode in sodium-ion chemistry.

The representative voltage potential profiles of the cycling stability test at the current density of 0.1 A g^−1^ are exhibited in Fig. 4c, where the cycle numbers are labelled on each curve. Upon the first sodiation process, we observed a major and a minor sodiation plateau at 0.5–0.2 V and 0.7–0.9 V. The minor plateau should be ascribed to the SEI film formation and responsible for the capacity loss at the beginning of the cycling stability tests. The discharge curves display a primary plateau at 0.4–0.2 V for the 2nd, 100th, and 500th cycle, and all charge curves (1st, 2nd, 100th, and 500th cycle) present a primary de-sodiation plateau at 0.4–0.6 V. The voltage potential profiles of the cycling stability tests at other current densities are depicted in Supplementary Fig. 10, in which the hysteresis between charging and discharging curves is slightly enlarged as the current rate increased. The rate performance and the cyclic voltammogram of the P_red_@CNF anode are exhibited in Supplementary Fig. 11 and 12. To investigate the role of SEI film formed with the FEC electrolyte additive, cells with and without FEC were tested at 0.1 A g^−1^ current densities, as shown in Supplementary Fig. 13. In contrast to cells without FEC additive, cells with FEC show a less severe capacity degradation and smaller efficiency fluctuation in 100 cycles, signifying that the stable long-term cycling performance necessitates the strong SEI films formed with FEC additive^36–38^.

### Post-cycling characterization

TEM image of the post-cycling P_red_@CNF anode after 5000 cycles at 1 A g^−1^ current density at de-sodiated state (Fig. 4d) shows that the P_red_ active material is still confined inside CNFs, indicating that the designed encapsulation strategy with core-shell nanostructure has successfully accommodated the volume variation of P_red_ particles, maintained the electronic contact between P_red_ cores and CNF shells and suppressed the side reactions consuming P_red_ active material. On the other hand, numerous bubbles are found in some of the post-cycling P_red_@CNF. Although the softening effect observed in the in situ TEM experiment (Fig. 3a) could be partially attributed to the heating effect caused by electron beam, bubbles found in the ex situ TEM image (formed by the shrinking of the softened P_red_ during the de-sodiation process) further exclude that hypothesis. The intercalation and extraction of alkali metal ions can amorphize anode materials, such as silicon and P_black_^39–41^, and thus the sodiation should be able to break the “chain-like” structure in the polymeric P_red_ into intermediate compounds with higher deformability. To investigate the intermediate phases of sodiated P_red_, Raman spectroscopy was performed on five sodiated P_red_@CNF samples, as shown in Supplementary Fig. 14. Realizing the complexity of the P–P covalent structure in phosphorus^42,43^, the in situ Raman spectroscopy is highly preferred here. However, due to the limitation of our equipment, we performed ex situ Raman experiments on the electrochemically sodiated samples by discharging electrodes to 0.05 V at 1 A g^−1^ current rate. In contrast to the spectrum of as-synthesized P_red_@CNF in Supplementary Fig. 14b, the P_red_ signal intensity of the five sodiated P_red_@CNF samples was greatly reduced in Supplementary Fig. 14a, as marked by the light red colour, indicative of the sodiation-induced P–P bond breaking and the formation of sodiated phosphorus compounds. In Supplementary Fig. 14a, the sodiated samples also presented a new band from 520 to 620 cm^−1^ (marked by the light blue colour), indicative of the intermediate phase formation, given the fact that P_white_ has characteristic Raman peaks at 367, 462, and 606 cm^−1^ ^44,45^.

## Discussion

For alloying anodes, such as silicon and tin, large volume variation always accompanies high capacity and high risk of material pulverization; and for silicon materials, a particle size of ~150 nm was believed to be the “no-fracture” size during lithiation^46^. Our observation showed that, instead of pulverization, the sodiation could result in the “liquid-like” behaviour of P_red_ particles, and thus P_red_ should have a much larger “no-fracture” size for sodium intercalation than that of silicon for lithium. The sodiation-induced softening of P_red_ reveals the superior malleability and deformability toward mitigating the adverse mechanical effects in an electrochemical battery system. Our study also suggests that, to further improve the electrochemical performance of P_red_-based anodes, part of the efforts should be devoted to the interface engineering at the electrode/electrolyte interfaces to avoid unfavourable side reactions, and the potential explorations could be conformal encapsulation and surface coating, artificial SEI films, and new electrolyte/additive combinations for even stronger SEI films.

## Conclusion

In summary, we investigated the sodiation process of synthesized P_red_-impregnated CNF composite through in situ TEM and chemo-mechanical simulation. The results of our study show that P_red_ particles can be softened during sodiation and thus it gains excellent malleability toward the high-capacity sodium-ion anodes. The fabricated P_red_@CNF anodes can deliver ~1850 mAh g^−1^ specific charge capacity over 500 cycles at 0.1 A g^−1^ current density, and >1000 mAh g^−1^ capacity over 5000 cycles at 1 A g^−1^ current rate. The excellent long-term cycling performance proves that the encapsulation strategy designed here can successfully suppress the unfavourable side reactions. Together, our studies provide an avenue to desirable sodium-ion anodes, and we hope that the data reported in this work will stimulate more theoretical and experimental efforts to explore phosphorus-based battery materials.

## Methods

### Materials preparation

The P_red_ was purchased from Spectrum Chemical Mfg. Corp. with 99.99% purity and the P_red_ powder was heated to 90 °C to remove the moisture. After drying, the powder was meshed in an argon-filled glovebox, using a mesh filter with 30 μm pore size. CNF powder (PR-XT-HHT from Pyrograf Products) was ultrasonicated in ethanol alcohol to break the ultra-long fibres and then annealed in argon flow at 1000 °C for 6 h to remove the moisture and unfavourable functional groups on both inner and outer surface of the CNFs. The P_red_ and CNF powder was mixed and filled into a 10-cm-long one-end-closed quartz tube in the glovebox, and the phosphorus/carbon weight ratio is 5:1. An ampoule was made by sealing the opening of the quartz tube under vacuum. In an argon-flowed tube furnace, the ampoule was heated at 550 °C for 30 min and then maintained at 280 °C overnight to convert existing P_white_ to P_red_. The ampoule was transferred into glovebox, and the product powder was taken out from the ampoule and transferred into an argon-flowed (350 sccm) tube furnace for the flash-heat treatment to remove the exceeding P_red_: the ceramic boat with product powder was first placed at the upstream region out of the heating zone, and then the boat was transferred into the heating zone for a flash-heat treatment at 600 °C for 90 s, and then the boat was maintained at 280 °C overnight with a cooling down process to get the final product. The resulted P_red_@CNF composite was assembled to a film through filtration in a glovebox, without any polymer binder or conductive carbon additive, and then the film was punched into discs with a diameter of 14 mm. The punched film discs were employed as electrodes directly in the subsequent electrochemical experiments. The mass of each P_red_@CNF film electrode is ~2.4 mg, and the loading mass of the P_red_ in each film electrode is ~0.84 mg according to the TGA results presented in Supplementary Fig. 4.

### Materials characterization

We examined surface morphology of synthesized samples using a JEOL 7001F-LV field-emission scanning electron microscope, with EDS module. We collected Raman spectra using a Renishaw inVia Raman Microscope, operating at 532 nm wavelength. The ex situ TEM, HAADF-STEM, and EDS element mapping analysis experiments were performed on a JEOL JEM 2100F field-emission (TEM), operating at 200 kV. In the ex situ TEM observation, synthesized sample powder was uniformly dispersed in solvents, such as ethanol, and then the mixture solution was dropped onto TEM grids (carbon-film-covered copper grids) and dried in an argon-filled glovebox. TGA was performed under a nitrogen atmosphere with a heating rate of 1 °C per minute, using a Netzsch Simultaneous Thermal Analyser.

### In situ TEM experiments

The in situ TEM electrochemical cell was incorporated into a Nanofactory TEM-STM specimen holder, in which the P_red_@CNF composite was dispersed onto a TEM half-grid with amorphous carbon support, as the active electrode material and current collector. Na metal was attached to a tungsten probe as the counter electrode, where a native Na_2_O layer formed on Na metal was employed as the solid electrolyte. Materials were assembled onto the specimen holder in an argon-filled glovebox, and then the assembly was transferred to the electron microscope using a sealed argon bag to prevent air exposure. The sodiation processes were recorded in real-time under TEM or STEM mode, using a TEM (JEOL 2100F), operating at 200 kV.

### Chemo-mechanical simulation

Considering the similarity between P_red_, amorphous silicon, and germanium, we simulate the sodiation of the P_red_ segment by a phenomenological two-step two-phase model. The sodiation is modelled by a nonlinear diffusion of Na with a diffusivity of *D* = *D*_0_[*C*_0_/(*C*_0_ − *C*) − 4*C*], where *D*_0_ is a diffusivity constant, *C* is the normalized Na concentration, and *C*_0_ is the local Na concentration required to break P–P bonds, and thus specifies the Na concentration of the intermediate phase during the two-step sodiation. Note that here the Na concentration is normalized by that of the fully sodiated phase Na_3_P; namely, *C* is zero represents pure phosphorus, while *C* is one represents Na_3_P. To illustrate the sodiation-induced deformation, an elastic and perfectly plastic model is adopted, with the total strain rate being a sum of three parts: $\dot \varepsilon _{ij} = \dot \varepsilon _{ij}^{\mathrm{e}} + \dot \varepsilon _{ij}^{\mathrm{p}} + \dot \varepsilon _{ij}^{\mathrm{s}}$ (*i* = 1, 2, 3 and *j* = 1, 2, 3). Here $\dot \varepsilon _{ij}^{\mathrm{e}}$ and $\dot \varepsilon _{ij}^{\mathrm{p}}$ present the elastic and plastic strain rate, respectively; $\dot \varepsilon _{ij}^{\mathrm{s}}$ presents the sodiation-induced volumetric strain rate and is related to the rate of normalized Na concentration $\dot C$ by $\dot \varepsilon _{ij}^{\mathrm{s}} = \beta _{ij}\dot C$, where $\beta _{ij}$ is the volume expansion coefficient caused by sodiation. The above nonlinear diffusion and elastic perfectly plastic model is numerically implemented in the finite element package ABAQUS to investigate the sodiation of P_red_ segments. In finite-element-based chemo-mechanical simulations, we take advantage of the axis-symmetric nature of the structure to reduce the numerical expanse. A CNF-encapsulated P_red_ segment is initially pristine and subjected to a constant Na flux *I*_0_ at its surface. The intermediate concentration *C*_0_ is set to be 0.3, considering the fact that P–P bond in P_red_ is weaker than Si–Si bonds in amorphous silicon (which corresponds to *C*_0_ = 0.67). The volume expansion coefficient *β*_*ij*_ (*i* = 1, 2, 3 and *j* = 1, 2, 3) is taken to be 0.462 to give a total volume increase of 300% at the formation of Na_3_P (note that the true volumetric strain is 0.462 at full sodiation and the associated engineering strain is 0.587). The yield stress *σ*_Y_ = 0.03*E*, where Young’s modulus *E* is taken to be 10 MPa for sodiated P_red_, and Poisson’s ratio ***v*** = 0.3. The CNF is modelled by shell elements with Young’s modulus of 50 GPa and Poisson’s ratio of 0.3. The mechanical interaction between the P_red_ segment and the CNF inner surface is described by a hard contact behaviour with a friction coefficient of 0.07.

### Electrochemical measurements

Using the P_red_@CNF film as working electrodes, all electrochemical measurements were carried out in half cells (CR2032-type coin cell) with sodium metal foils as counter electrodes, and 1.0 mol L^−1^ NaPF_6_ (Sigma-Aldrich, >99%) was mixed with EC–DEC as the electrolyte. Targeting a LiF-enriched SEI film with good electrochemical and mechanical stability, FEC was added into the electrolyte (10%, by volume) as additive. Before assembling electrodes into coin cells, all electrodes were fully immersed in electrolyte overnight (at least 12 h). All cells were assembled inside an argon-filled glovebox. Battery cells were tested with the voltage window of 0.01–1.5 V vs. Na/Na^+^. In order to optimize the cycling performance, for the first three cycles, anodes were cycling at 0.1 A g^−1^ current density to form stable SEI layers at the earliest stage of the cycling test. All of the capacity and current density volumes were calculated from the mass of phosphorus only. For the ex situ Raman experiments, the as-prepared P_red_@CNF film electrodes were assembled into battery cells and discharged to 0.05 V at 1 A g^−1^ current rate. The sodiated electrodes were taken out from the disassembled cells and washed with electrolyte solvent briefly in a glovebox, and then sealed between two glass slides for Raman spectroscopy investigation.

## Supplementary information

Supplementary Information

Description of Additional Supplementary Files

Supplementary Movie 1

Supplementary Movie 2

## Data Availability

All data generated in this study and supported the findings of this work are available from the corresponding author on reasonable request.

## Code availability

The code employed in this study and supported the findings of this study are available from the corresponding author on reasonable request.
