# Supplementary material for: Red-phosphorus-impregnated carbon nanofibers for sodium-ion batteries and liquefaction of red phosphorus
Source: Nat Commun. 2020 May 20;11:2520. doi: 10.1038/s41467-020-16077-z (PMC7239945; doi:10.1038/s41467-020-16077-z)
Supplement: Supplementary file 1 — Supplementary Information [file 41467_2020_16077_MOESM1_ESM.pdf]

Supplementary Information

Red-Phosphorus-Impregnated Carbon Nanofibers for Sodium-Ion Batteries and Liquefaction of Red Phosphorus

Liu et al.

Supplementary Figures

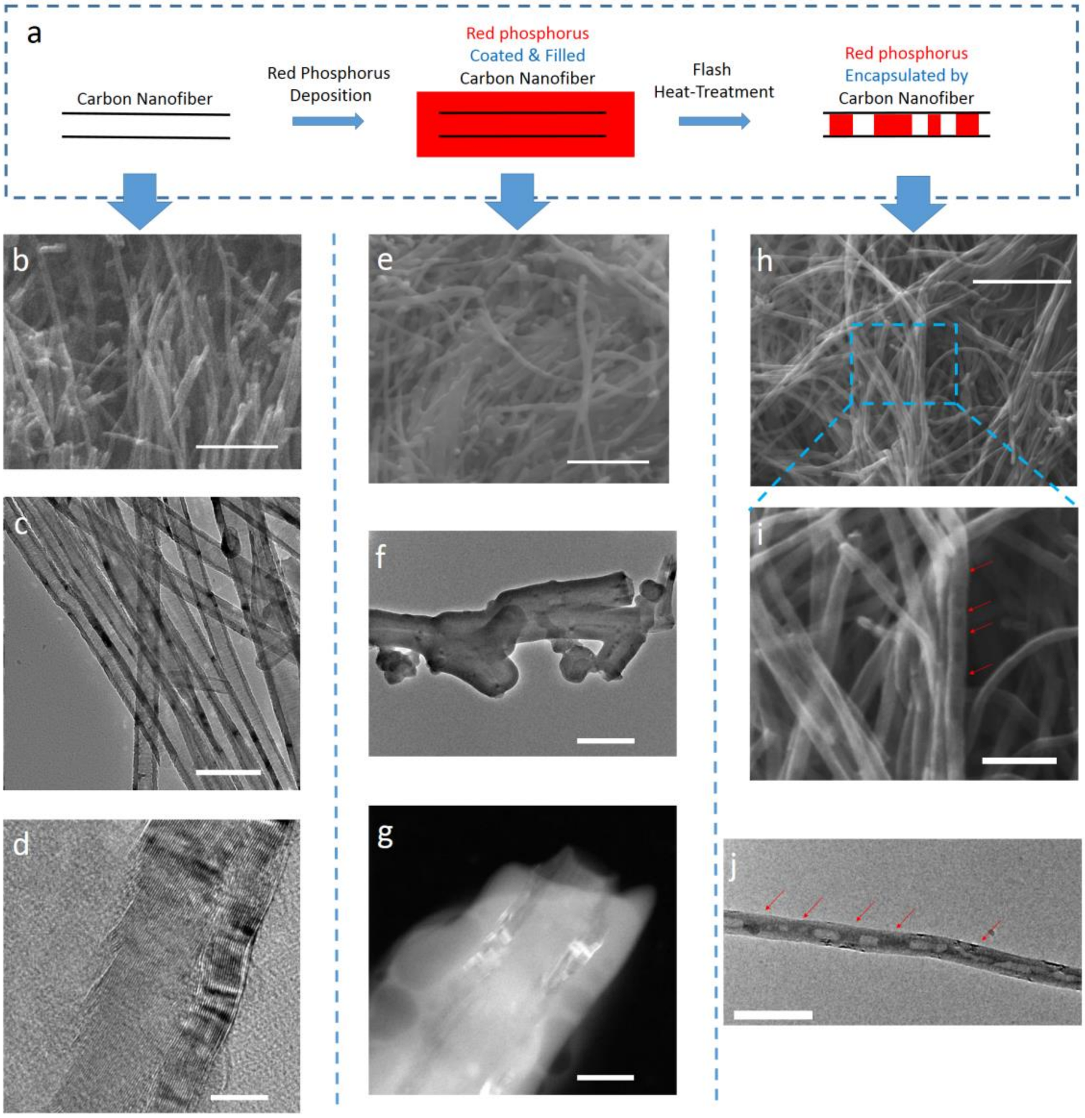

Supplementary Figure 1. Synthesis route and morphology characterizations of the P_{red}@CNF in different stages. (**a**) Schematic description of the synthesis process flow of carbon nanofiber impregnated red phosphorus (P_{red}@CNF). (**b, c, d**) SEM, TEM and high-resolution TEM image of CNF. Scale bars are 1 μm, 500 nm, and 20 nm for (**b**), (**c**), and (**d**), respectively. (**e, f, g**) SEM, TEM, and STEM image of P_{red} coated CNF, Scale bars are 1 μm, 200 nm, and 100 nm for (**e**), (**f**), and (**g**), respectively. (**h, i, j**) SEM and TEM image of P_{red}@CNF, where (**i**) is the enlarged SEM image of the area marked with the blue dashed rectangle in (**h**), and P_{red} segments are indicated by the red arrows in (**i**) and (**j**). Scale bars are 1 μm for (**h**), (**i**), and (**j**).

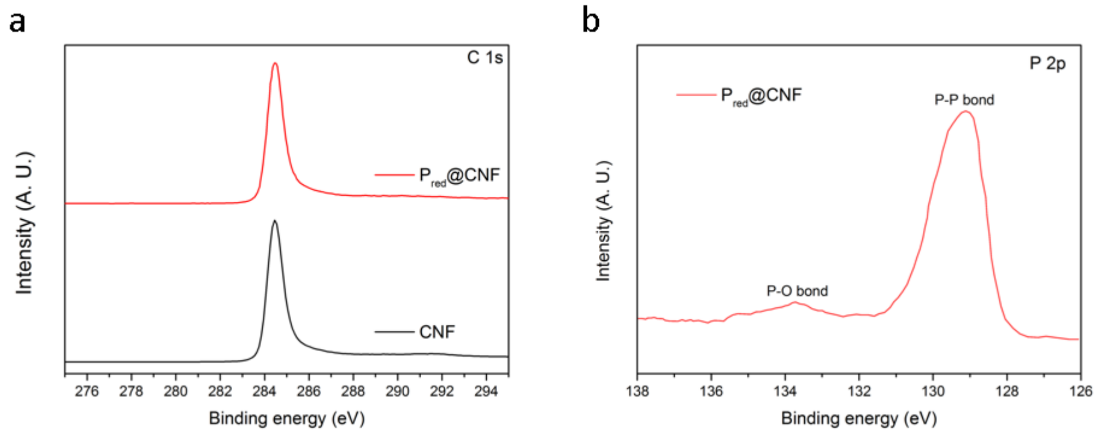

Supplementary Figure 2. XPS profiles of CNF and P_{red}@CNF. (**a**) High-resolution XPS spectrum of carbon 1s of the CNF and synthesized P_{red}@CNF. (**b**) High-resolution XPS spectrum of phosphorus 2p of the synthesized P_{red}@CNF, where the P-O bond signal may come from the air exposure of the samples during the transfer process from glovebox to XPS facility.

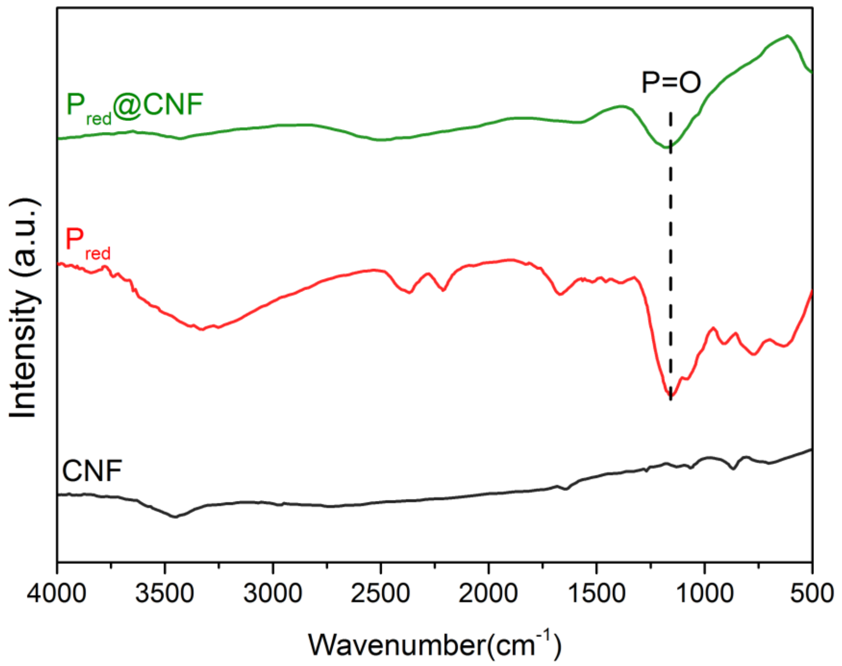

Supplementary Figure 3. FTIR profiles of the CNF, P_{red}, and as-synthesized P_{red}@CNF.

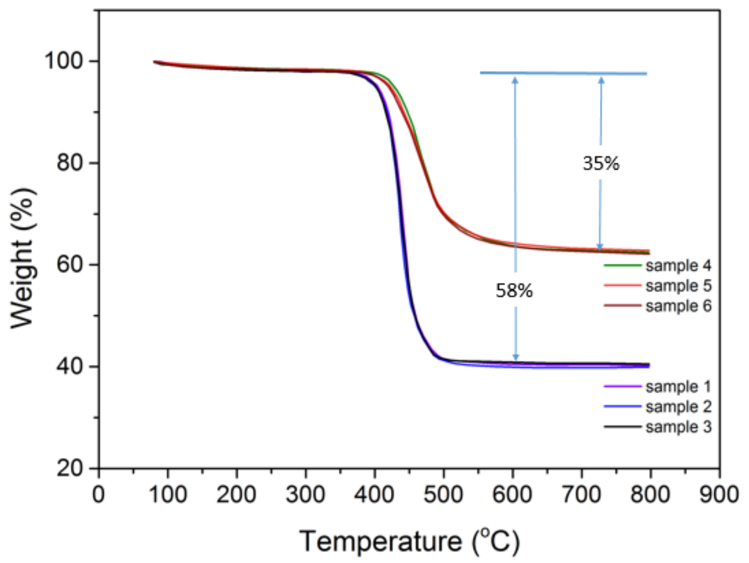

Supplementary Figure 4. Thermogravimetric analysis (TGA) of three P_{red}/CNF samples and three P_{red}@CNF samples. The P_{red}/CNF samples (before flash-heat-treatment) in three different bunches are numbered from 1 to 3 and the P_{red}@CNF samples (after flash-heat-treatment) in three different bunches are numbered from 4 to 6.

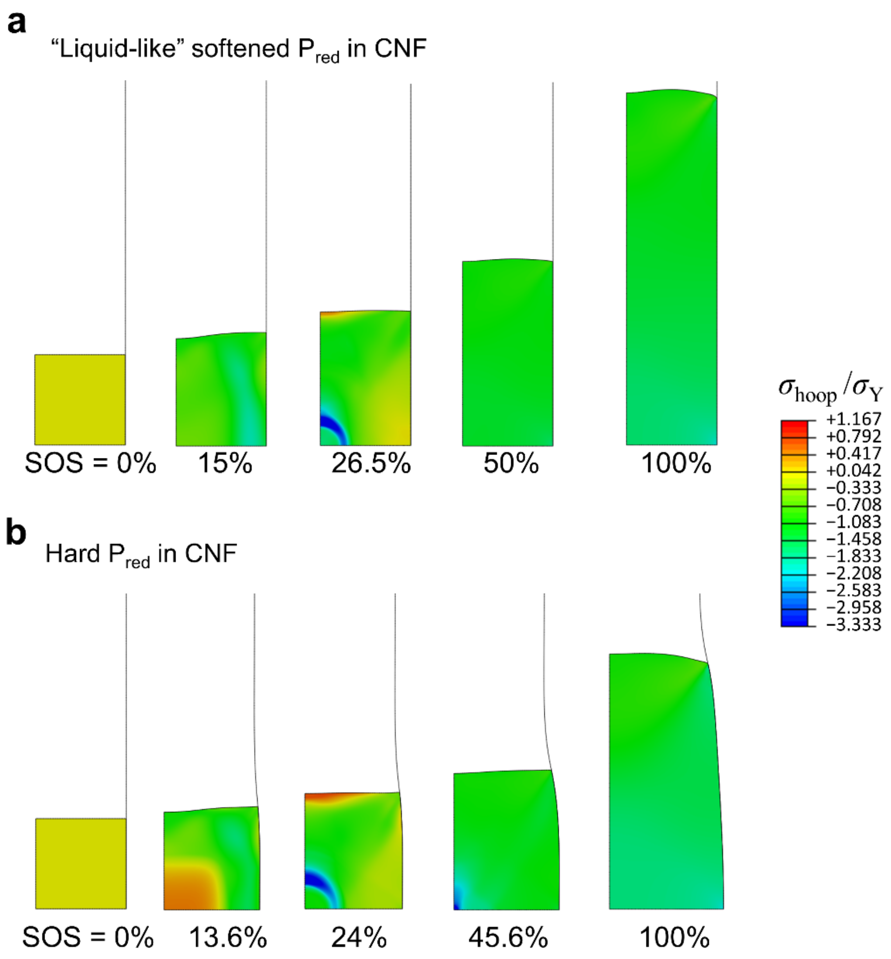

Supplementary Figure 5. Simulation of the sodiation process on soft and hard P_{red} segments. (**a**) Simulated morphology change of a sodiation-softened P_{red} segment (with Young's modulus of 10 MPa) during the different states of sodiation (SOS). The softened P_{red} segment flows readily in the CNF without apparently deforming the CNF, in good agreement with the experimental observation. (**b**) Simulated morphology change of a hard P_{red} segment with Young's modulus of 10 GPa during sodiation. The hard segment without sodiation-induced softening significantly expands the CNF laterally, which is in stark contrast to the experimental observation.

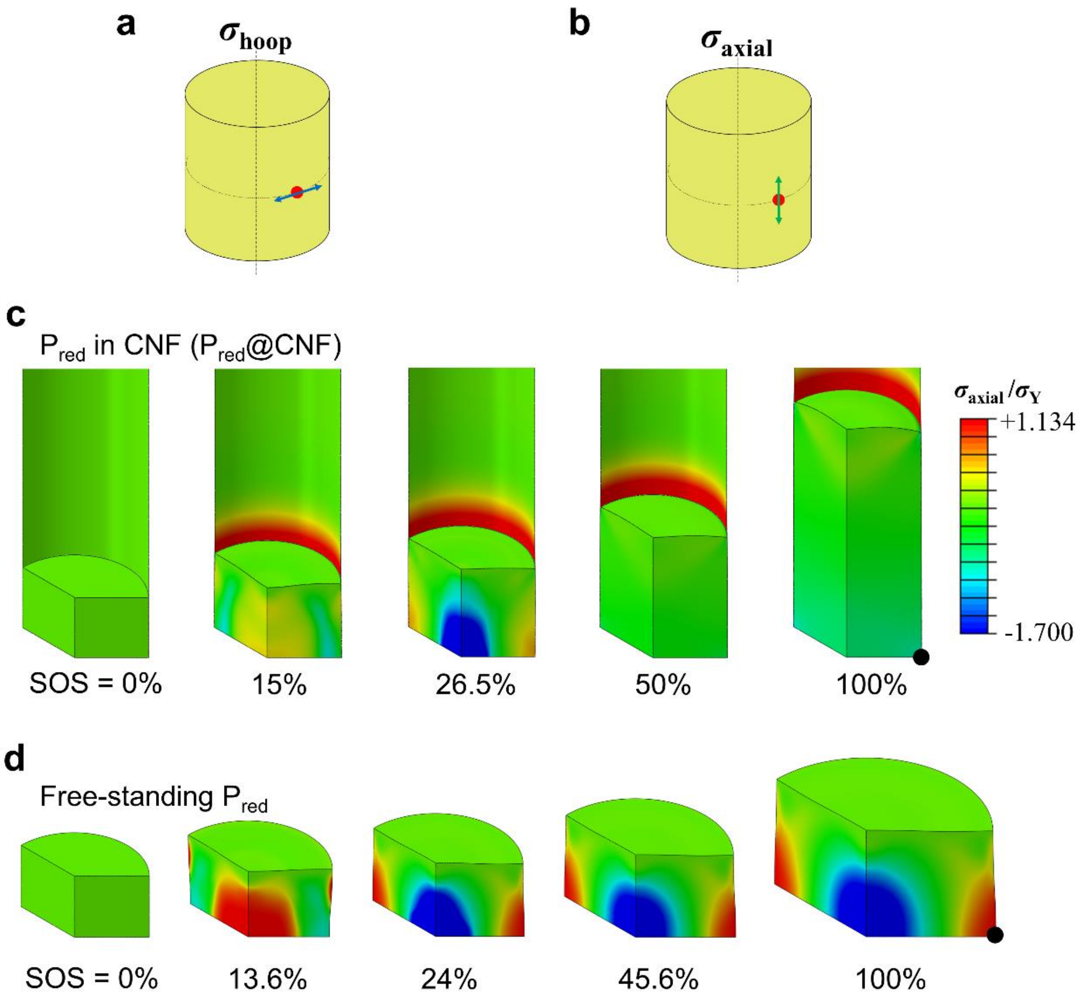

Supplementary Figure 6. Simulation results of the hoop and axial stresses. (**a**) Illustration of the hoop stress σ_{hoop}. (**b**) Illustration of the axial stress σ_{axial}. (**c**) Simulated evolution of axial stress in a CNF-encapsulated P_{red} segment from SOS=0% to SOS=100%. (**d**) Simulated evolution of axial stress in a free-standing P_{red} segment from SOS=0% to SOS=100%.

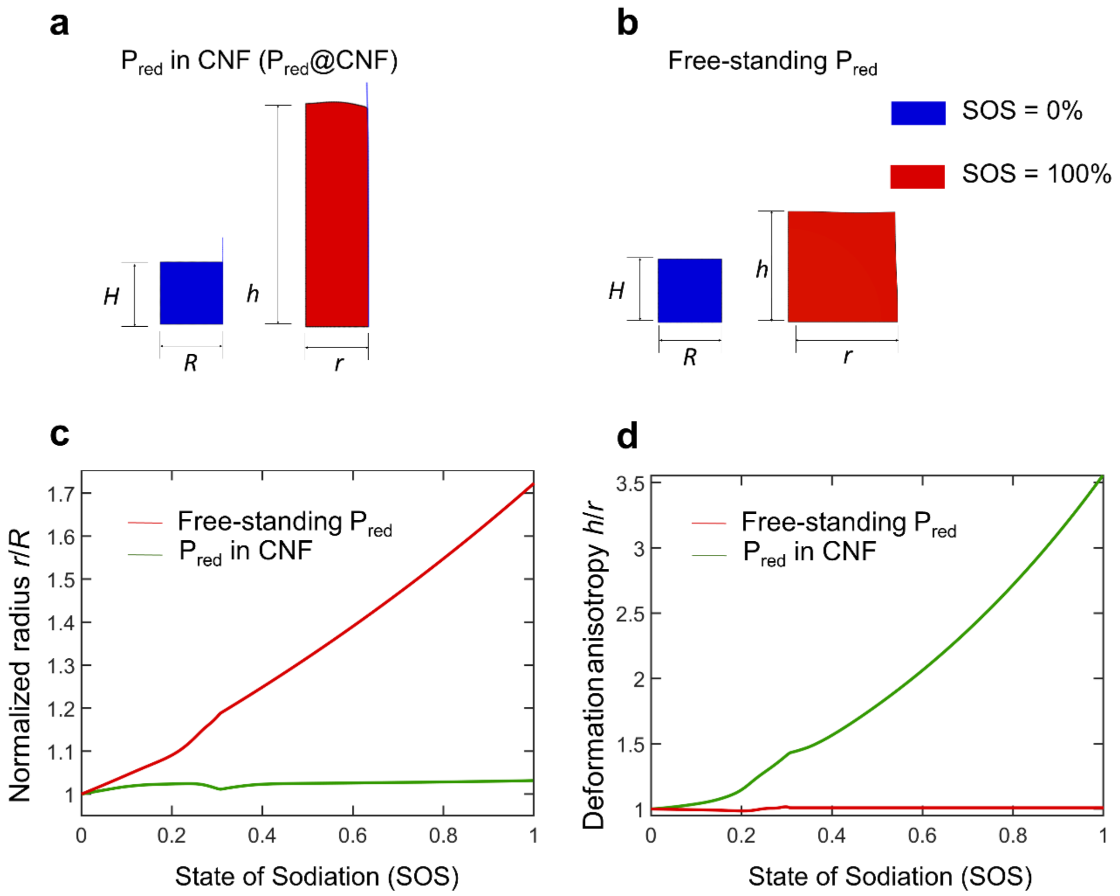

Supplementary Figure 7. Simulation results of the volumetric expansion. (a) Simulated anisotropic expansion of CNF-encapsulated P_{red} (P_{red}@CNF). The P_{red} segment with sodiation-induced mechanical softening flows readily along the longitudinal direction of the CNF, leaving the CNF almost without any deformation in the radial direction. The original height and radius of the P_{red} segment are given by *H* and *R*, respectively, and the height and radius of P_{red} after sodiation are represented by *h* and *r*, respectively. (**b**) Simulated isotropic expansion of a free-standing P_{red} segment. (**c**) The radial deformation of the CNF-encapsulated P_{red} (P_{red}@CNF) and free-standing segments during the sodiation process. (**d**) The axial deformation of CNF-encapsulated P_{red} (P_{red}@CNF) and free-standing segments during the sodiation process.

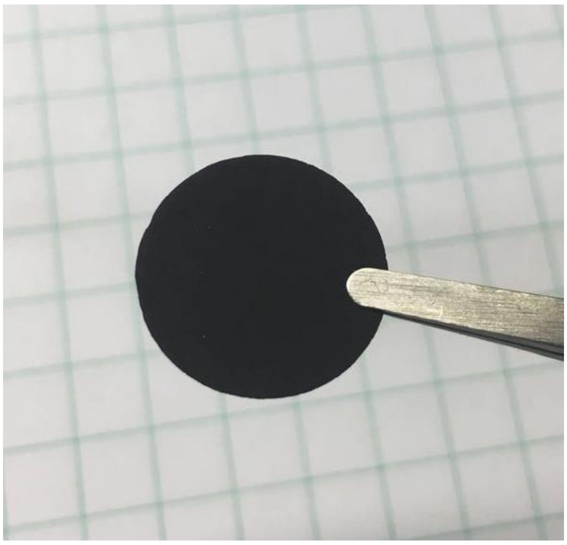

Supplementary Figure 8. Optical image of the fabricated binder- and additive-free film electrode through vacuum filtration. The film has been punched into a disc with a diameter of 14 mm.

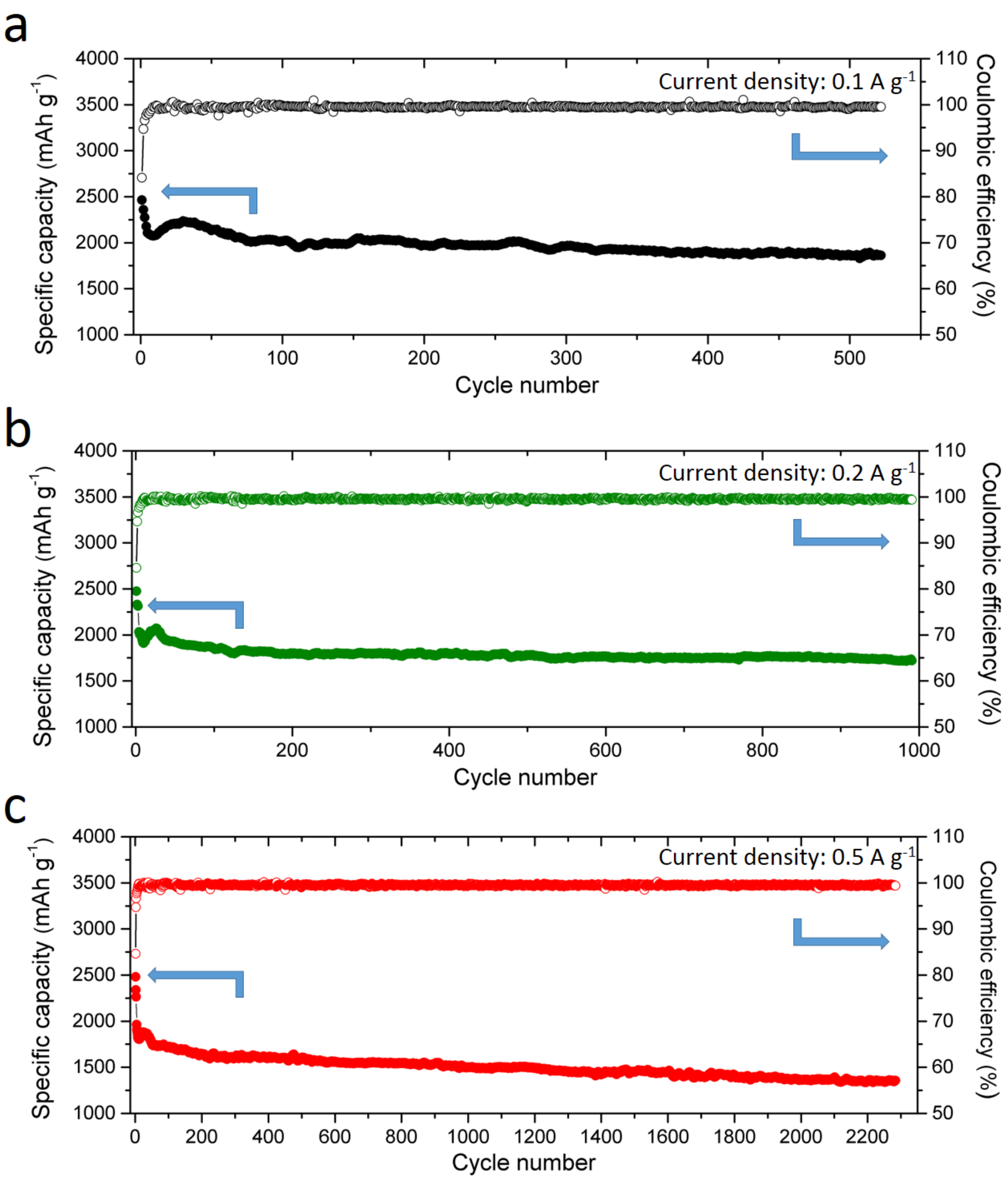

Supplementary Figure 9. The cycling performance of P_{red}@CNF anodes. The current densities are (**a**) 0.1, (**b**) 0.2, and (**c**) 0.5 A g^{-1}, with cycle numbers up to 522, 1000, and 2284, marked with different colours, respectively. The Coulombic efficiency is plotted as open circles against the right-hand axis. In (**b**) and (**c**), anodes were cycled at 0.1 A g^{-1} current density for the first three cycles, and then the current densities were changed to 0.2 and 0.5 A g^{-1}, respectively.

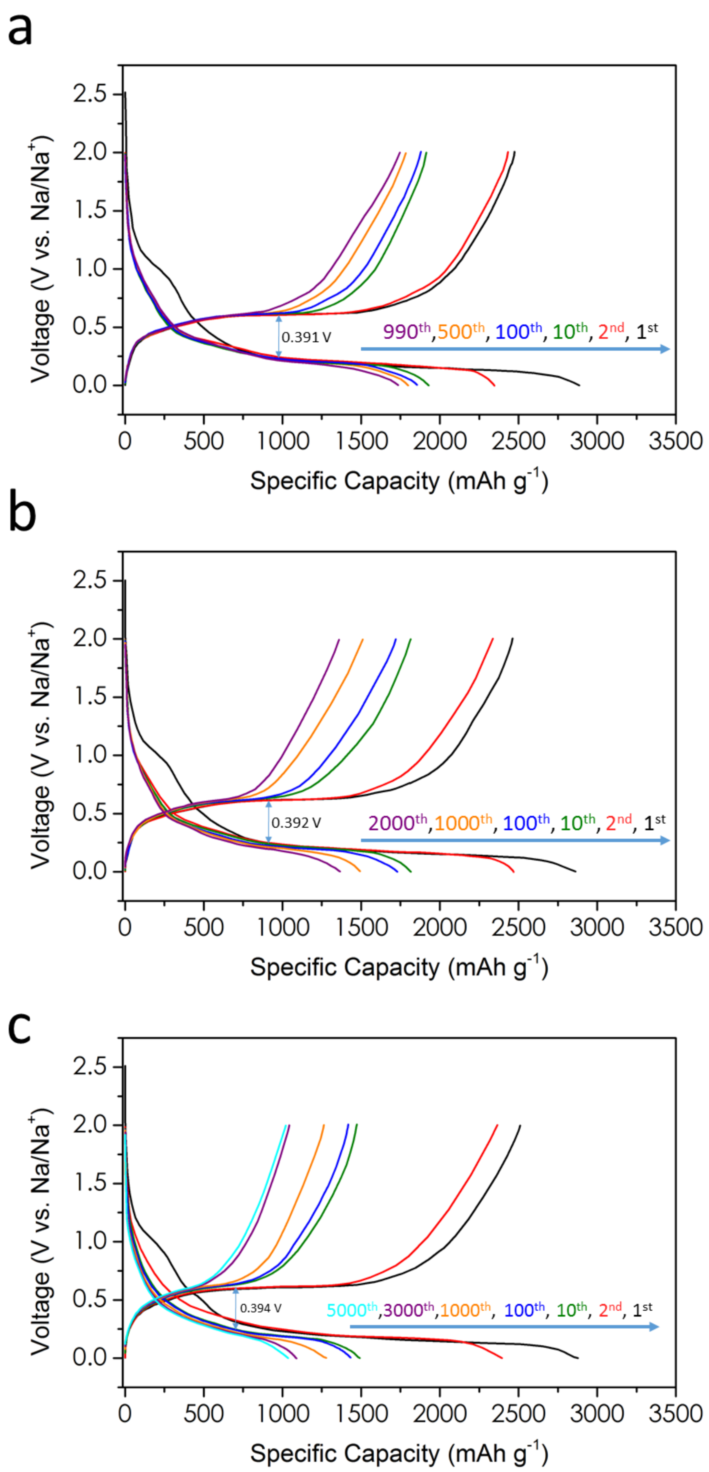

Supplementary Figure 10. Representative voltage profiles of the cycling performance. The current densities are (**a**) 0.2, (**b**) 0.5, and (**c**) 1 A g^{-1}. All anodes were cycled at 0.1 A g^{-1} current density for the first three cycles. Potential hysteresis between charging and discharging curves was measured at the half-capacity points of the 10th cycle.

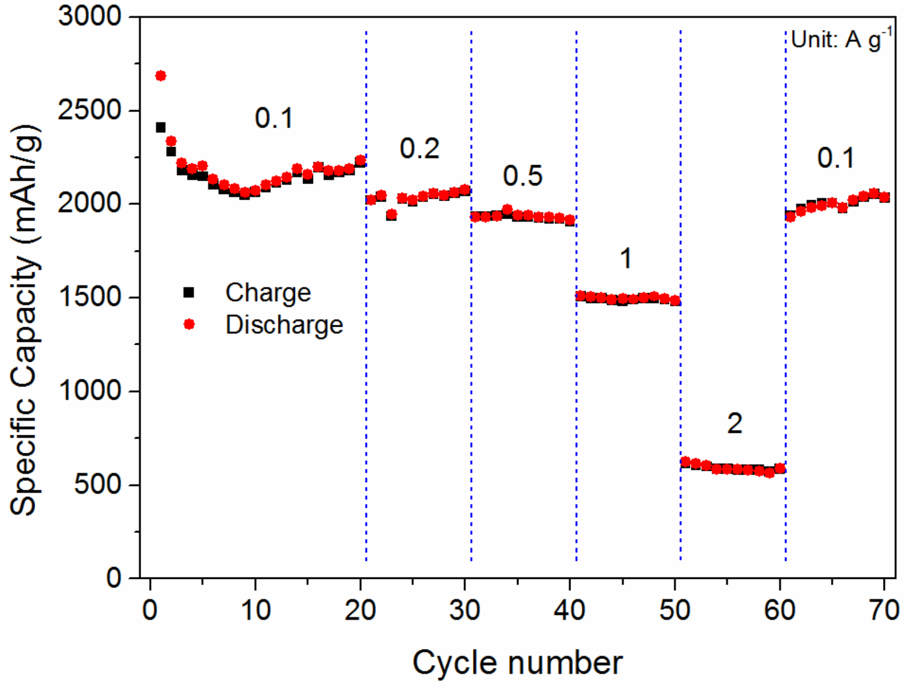

Supplementary Figure 11. Rate performance of the P_{red}@CNF anode. The current density was increased from 0.1 to 2 A g^{-1}, and then switched back to 0.1 A g^{-1}.

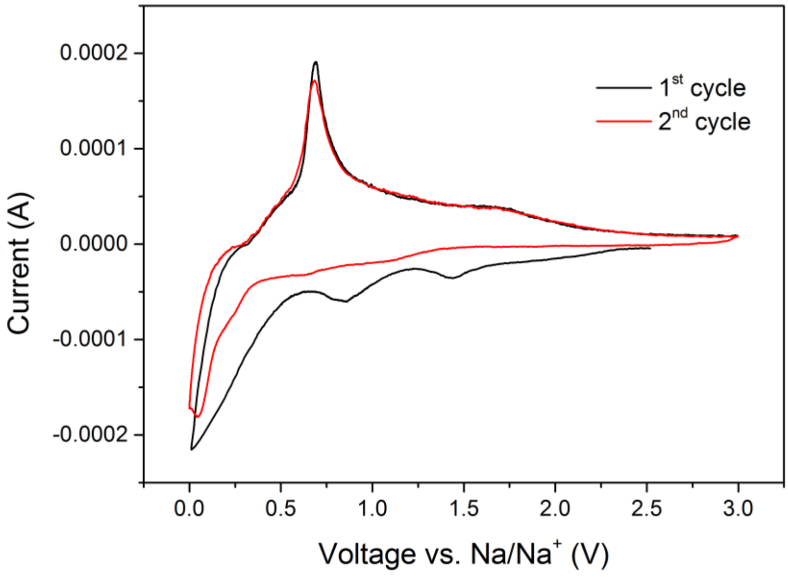

Supplementary Figure 12. Cyclic voltammogram of the P_{red}@CNF anode.

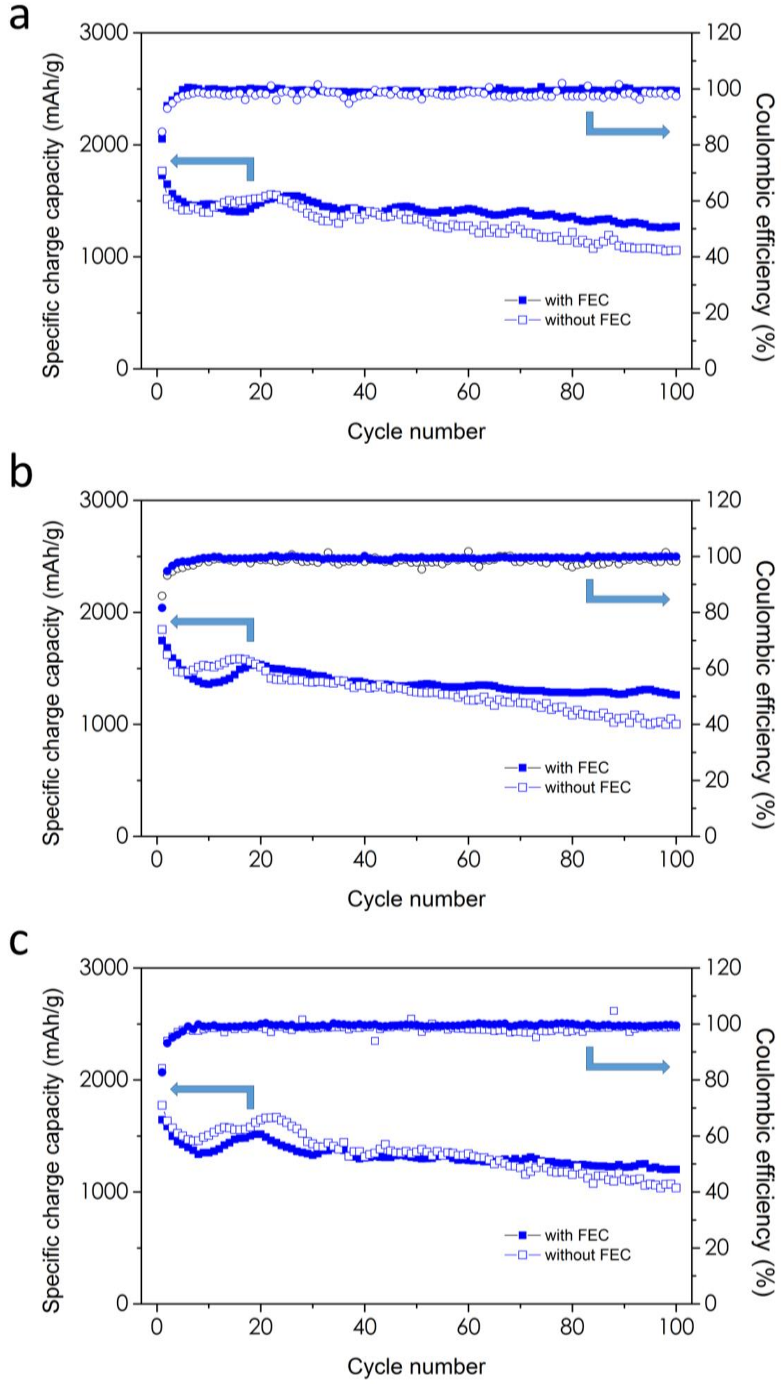

Supplementary Figure 13. The cycling performance of three groups of cells with and without FEC electrolyte additive. The current density is 0.1 A g^{-1}.

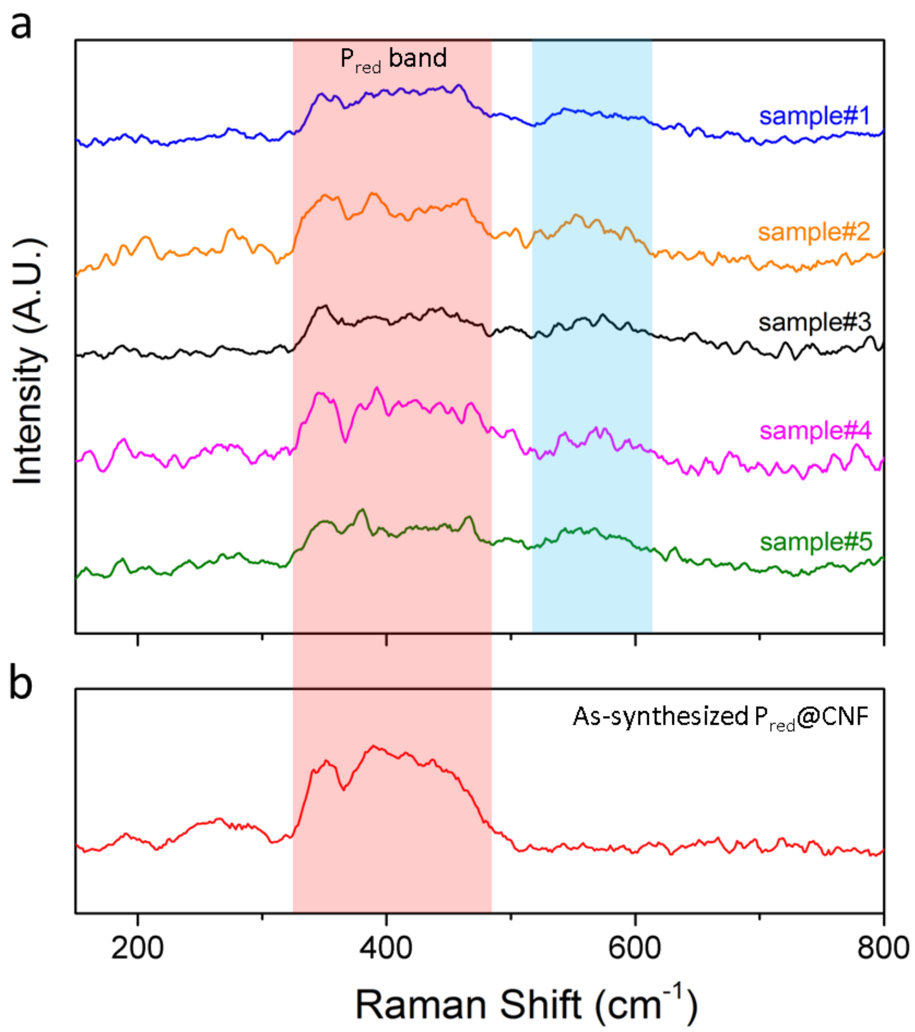

Supplementary Figure 14. Raman spectra of sodiated and as-synthesized P_{red}@CNF. (**a**) Spectra of five sodiated samples and (**b**) as-synthesized P_{red}@CNF as control. The light red rectangular box marks the Raman band of red phosphorus, and the light blue rectangular box indicates the emerging of the new band, indicative of the intermediate phase formation.

Supplementary Note

As illustrated in Supplementary Fig. 1, P_{red}@CNF was synthesized through the vaporization-condensation method. First, P_{red} was deposited onto and into the CNFs in a sealed quartz ampoule under vacuum, and then the exceeding P_{red} was removed in an argon-flowed tube furnace with a flash-heat-treatment process, as the process flow described in Supplementary Figure 1a. Carbon nanofiber (CNF) was characterized by SEM and TEM, as shown in Supplementary Fig. 1b to 1d. In Supplementary Fig. 1b and 1c, the length of most CNFs ranges from 1 to 5 μm, and the diameter is around 150 nm. The high-resolution TEM image shows that the wall thickness of CNF is around 40 nm. After the P_{red} deposition, the P_{red}/CNF composite was characterized by SEM and TEM, as shown in Supplementary Fig. 1e to 1g. In contrast to the SEM image of CNF in Supplementary Fig. 1b, the P_{red} coated CNF is not transparent anymore in Supplementary Fig. 1e, and the welding between P_{red}/CNFs fibers indicates that P_{red} has been deposited onto CNFs. The P_{red} deposition on CNFs can be observed more clearly in the TEM and STEM images exhibited in Supplementary Fig. 1f and 1g, showing the uniform coating of P_{red} on CNFs. After the flash-heat-treatment process, the final product was characterized by SEM and TEM, as shown in Supplementary Fig. 1h to 1j. In Supplementary Fig. 1h, the SEM image shows that the CNFs become transparent again, indicating the exceeding P_{red} coated on the outside of CNFs has been removed. The P_{red} segments in most of the CNFs are visible under SEM at 15k eV acceleration voltage, as shown by the red arrows in the

enlarged SEM image exhibited in Supplementary Fig. 1i, indicative of the good uniformity of the synthesized P_{red}@CNF composite. The TEM image in Supplementary Fig. 1j shows a single CNF with several P_{red} segments inside.
