# Supplementary material for: Red-phosphorus-impregnated carbon nanofibers for sodium-ion batteries and liquefaction of red phosphorus
Source: Nat Commun. 2020 May 20;11:2520. doi: 10.1038/s41467-020-16077-z (PMC7239945; doi:10.1038/s41467-020-16077-z)
Supplement: Supplementary file 2 — Description of Additional Supplementary Files [file 41467_2020_16077_MOESM2_ESM.pdf]

Description of Additional Supplementary Files

File Name: Supplementary Movie 1
Description: Sodiation process of four red phosphorus segments inside a single carbon nanofiber.

File Name: Supplementary Movie 2
Description: Two red phosphorus segments deformed and merged together during the sodiation expansion.
